# Combined Targeting of Estrogen Receptor Alpha and Exportin 1 in Metastatic Breast Cancers

**DOI:** 10.3390/cancers12092397

**Published:** 2020-08-24

**Authors:** Eylem Kulkoyluoglu Cotul, Qianying Zuo, Ashlie Santaliz-Casiano, Ozan Berk Imir, Ayca Nazli Mogol, Elif Tunc, Kevin Duong, Jenna Kathryn Lee, Rithva Ramesh, Elijah Odukoya, Mrinali P. Kesavadas, Monika Ziogaite, Brandi Patrice Smith, Chengjian Mao, David J. Shapiro, Ben Ho Park, Benita S. Katzenellenbogen, Drew Daly, Evelyn Aranda, John D. O’Neill, Christopher Walker, Yosef Landesman, Zeynep Madak-Erdogan

**Affiliations:** 1 Department of Food Science and Human Nutrition, University of Illinois at Urbana-Champaign, Urbana, IL 61801, USA; ekulkoyl@purdue.edu (E.K.C.); qzuo2@illinois.edu (Q.Z.); ekardiat@gmail.com (E.T.); jklee12@illinois.edu (J.K.L.); mrinalipk@gmail.com (M.P.K.); 2 Division of Nutritional Sciences, University of Illinois at Urbana-Champaign, Urbana, IL 61801, USA; ashlies2@illinois.edu (A.S.-C.); imir2@illinois.edu (O.B.I.); amogol2@illinois.edu (A.N.M.); 3 Department of Molecular and Cellular Biology, University of Illinois at Urbana-Champaign, Urbana, IL 61801, USA; kevin.duong@uth.tmc.edu (K.D.); rrames6@illinois.edu (R.R.); 4 Department of Human Development and Family Studies, University of Illinois at Urbana-Champaign, Urbana, IL 61801, USA; lileli1354@yahoo.com; 5 Department of Interdisciplinary Health Sciences, University of Illinois at Urbana-Champaign, Urbana, IL 61801, USA; mziogaite@gmail.com; 6 Department of Informatics, University of Illinois at Urbana-Champaign, Urbana, IL 61801, USA; brandis2@illinois.edu; 7 Department of Biochemistry, University of Illinois at Urbana-Champaign, Urbana, IL 61801, USA; cmao@illinois.edu (C.M.); djshapir@illinois.edu (D.J.S.); 8 Cancer Center at Illinois, Urbana, IL 61801, USA; katzenel@illinois.edu; 9 Division of Hematology and Oncology, Vanderbilt University Medical Center, Nashville, TN 37232, USA; ben.h.park@vumc.org; 10 Department of Molecular and Integrative Physiology, University of Illinois at Urbana-Champaign, Urbana, IL 61801, USA; 11 Xylyx Bio, Inc., Brooklyn, NY 11226, USA; drew@xylyxbio.com (D.D.); evelyn@xylyxbio.com (E.A.); john@xylyxbio.com (J.D.O.); 12 Karyopharm Therapeutics, Newton, MA 02459, USA; christopher.walker@karyopharm.com (C.W.); ylandesman@karyopharm.com (Y.L.); 13 Beckman Institute for Advanced Science and Technology, Urbana, IL 61801, USA; 14 Carl R. Woese Institute of Genomic Biology, Urbana, IL 61801, USA

**Keywords:** breast cancer, *ESR1* mutant models, combination therapies, metabolic rewiring, glutamine, tamoxifen, selinexor

## Abstract

The majority of breast cancer specific deaths in women with estrogen receptor positive (ER^+^) tumors occur due to metastases that are resistant to therapy. There is a critical need for novel therapeutic approaches to achieve tumor regression and/or maintain therapy responsiveness in metastatic ER^+^ tumors. The objective of this study was to elucidate the role of metabolic pathways that undermine therapy efficacy in ER^+^ breast cancers. Our previous studies identified Exportin 1 (XPO1), a nuclear export protein, as an important player in endocrine resistance progression and showed that combining selinexor (SEL), an FDA-approved XPO1 antagonist, synergized with endocrine agents and provided sustained tumor regression. In the current study, using a combination of transcriptomics, metabolomics and metabolic flux experiments, we identified certain mitochondrial pathways to be upregulated during endocrine resistance. When endocrine resistant cells were treated with single agents in media conditions that mimic a nutrient deprived tumor microenvironment, their glutamine dependence for continuation of mitochondrial respiration increased. The effect of glutamine was dependent on conversion of the glutamine to glutamate, and generation of NAD^+^. PGC1α, a key regulator of metabolism, was the main driver of the rewired metabolic phenotype. Remodeling metabolic pathways to regenerate new vulnerabilities in endocrine resistant breast tumors is novel, and our findings reveal a critical role that ERα-XPO1 crosstalk plays in reducing cancer recurrences. Combining SEL with current therapies used in clinical management of ER^+^ metastatic breast cancer shows promise for treating and keeping these cancers responsive to therapies in already metastasized patients.

## 1. Introduction

The nuclear hormone receptor, estrogen receptor alpha (ERα), is present in approximately 70% of both early and late stage human breast cancers (BCas) [1,2]. ERα is targeted by endocrine therapies, which are well tolerated and provide long-term disease free survival if patients have localized disease [1]. Unfortunately, 10–40% of patients with ER^+^ disease experience recurrence and metastasis within 20 years [3,4,5,6,7]. The recurrence of cancer in ER^-^ patients is higher in the first five years after the diagnosis, yet for ER^+^ patients there is a substantial long-term risk of death due to metastatic BCa even 20 years after the initial diagnosis [8]. The 5-year relative survival rate of patients with ER^+^ metastatic disease is 24%, almost none are cured, and each year nearly 28,000 women in the United States with recurrent ER^+^ metastatic tumors die [2,3,9].

Extended endocrine therapy combined with agents, such as cyclin dependent kinase (CDK) inhibitors or mammalian target of rapamycin (mTOR) inhibitors is the most current treatment option for ER^+^ metastatic cancer [2]. Yet therapy-resistance develops during the course of initial and subsequent treatment in almost all patients due to alterations of sequence or copy number for critical genes such as *ESR1*, *PIK3CA* or *CCND1*, signaling pathways such as HER2, PI3K or FGF signaling, and drug uptake and metabolism [5,10]. Thus, acquired therapy resistance remains a significant clinical problem that indicates a critical need for therapeutic approaches by which a sustained regression of ER^+^ metastatic tumors can be achieved. Identification of pathways that promote therapy resistance and metastasis provide an opportunity for developing more effective therapy regimens.

In our previously published studies, we identified a group of nuclear transport proteins, including XPO1, which are upregulated in tamoxifen resistant cell lines and tumors. XPO1 up-regulation caused nuclear export of factors that are required in the nucleus for ERα-dependent transcriptional responses [11,12]. We previously showed that XPO1 mRNA and protein expression are higher in Luminal B subtype of tumors, which are more refractory to endocrine treatments [11,13]. High *XPO1* mRNA expression is associated with a poor outcome in women who are treated with tamoxifen (TAM) [11]. In cells that are resistant to the active form of TAM, 4-hydroxy-tamoxifen (4-OHT), combined targeting of ERα and XPO1 prevented 4-OHT induced cell proliferation and anchorage independent growth [11], and induced autophagy [13]. In BT474 tumor xenografts, TAM stimulated tumor growth whereas SEL inhibited growth. However, tumors that regressed with SEL only treatment, came back within 3 weeks after treatments were stopped [11]. The combination of SEL and TAM not only caused a faster and more complete regression of tumors, but also the regression was sustained even a month after the administration of SEL and TAM was stopped. In our follow-up studies, we showed that XPO1 induced endocrine resistance by modulating PI3K/Akt signaling and increased survival of BCa cells by increasing mitochondrial and glycolytic respiration. Combined ERα and XPO1 targeting inhibited activation of cell survival mechanisms to provide sustained tumor regression [13]. However, we do not know if we can combine XPO1 inhibitors with other therapies currently used in the clinics for treatment of metastatic ER^+^ BCas.

In this study, we focused on ER^+^ tumors with *ESR1* mutations, which comprise about 30−40% of all patients with ER^+^ metastatic breast cancer (MBC). We elucidated the causes and mechanisms of therapy resistance by focusing on the role of metabolic changes in tumor cells to adapt to and survive in metastatic environments in the presence of treatments. We tested the therapeutic potential of combining XPO1 inhibitors with current clinical drugs to prevent therapy resistance during metastatic tumor treatment. Our studies validated XPO1 as a target, whose co-inhibition together with ERα would enhance the effectiveness of current therapies, by preventing metabolic adaptation and improved survival in metastatic organ sites during sequential therapies.

## 2. Results

### 2.1. Clinical Relevance of Combining XPO1 Inhibitors with Current Therapies in Metastatic ER^+^ Tumors: Impact on Metastasis Related Gene Expression and Therapy Resistant Cells

**Figure 1:**
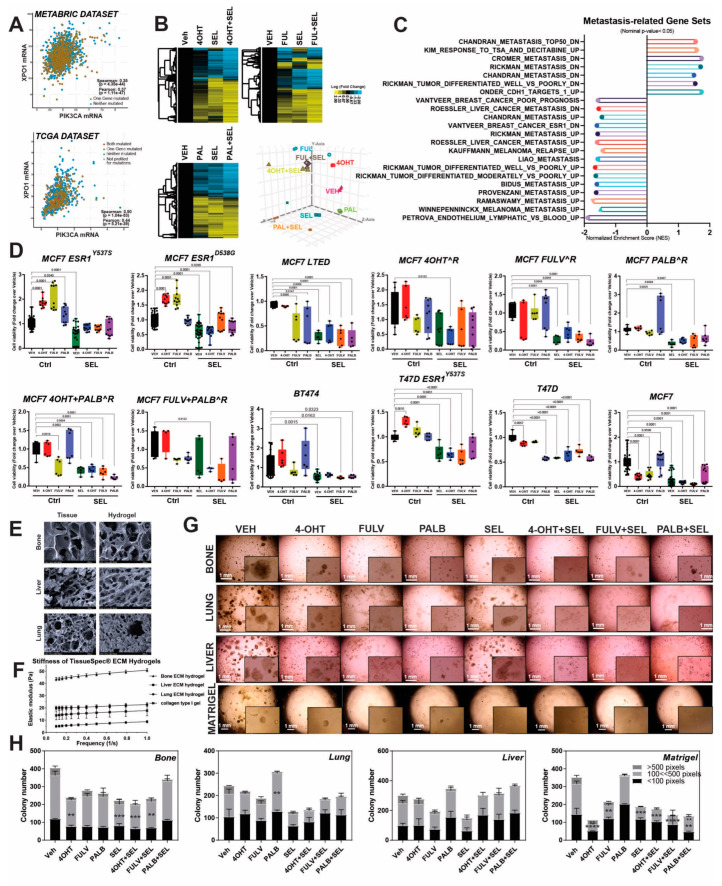
Clinical relevance of combining XPO1 inhibitors with current therapies in metastatic ER^+^ tumors: Impact on metastasis related gene expression and therapy resistant cells. (**A**) Correlation between *XPO1* and *PIK3CA* expression in ER^+^ tumors in METABRIC and TCGA datasets. Spearman and Pearson correlation analyses coefficients and *p*-values were indicated for each dataset (METABRIC data set: Spearman correlation = 0.35, Pearson correlation = 0.37, TCGA data set: Spearman correlation = 0.5, Pearson correlation = 0.44). (**B**) RNA-Seq analysis in MCF7-*ESR1^Y537S^* cells, treated with 1 µM 4-OHT, Fulv or Palb in the presence or absence of 100 nM SEL. PCA plot showing distinct gene expression patterns with the different treatments are shown at the right bottom panel of (B). (**C**) Gene-set enrichment analysis (GSEA) of gene sets that were enriched in 4-OHT + SEL dataset. (**D**) Cell viability assay in different endocrine-sensitive or endocrine-resistant breast cancer cell lines (MCF7 Parental, MCF7-*ESR1^Y537S^*, MCF7-*ESR1^D538G^*, T47D Parental, T47D-*ESR1^Y537S^*, T47D-*ESR1^D538G^*, MCF7 cells treated with different treatments and BT474), cotreated with 4-OHT [10^−6^ M], Fulv [10^−6^ M] and Palb [10^−6^ M] and SEL [10^−7^ M]. A one-way analysis of variance (ANOVA) model was used for statistical significance of change in cell viability with different treatments. All values were presented as mean ± SEM from six independent repeats. (**E**) Scanning electron microscopy (SEM) images of human tissues and decellularized tissue from which ECM hydrogels are derived. (**F**) Stiffness of TissueSpec^®^ ECM Hydrogels. (**G**). MCF7-*ESR1^Y537S^* cells were cultured at a density of 2 × 10^3^ cells/well and embedded in 3D hydrogels to mimic different metastatic niches (bone, liver, lung) for breast cancer cells, and they were treated with different endocrine agents (4OHT [10^−6^], Fulv [10^−6^] and Palb [10^−6^]) and in combination with SEL [10^−7^]. (**H**). Colony size (pixels) and number were quantified using OpenCFU colony counting software (http://opencfu.sourceforge.net/). A one-way analysis of variance (ANOVA) model was used for statistical significance of treatment and values were presented as mean ± SEM from three biological replicates (** *p* <0.01, *** *p* <0.001; **** *p* < 0.0001).

To assess viability of XPO1 as a therapeutic target in ER^+^ metastatic tumors, we utilized TCGA and METABRIC datasets and studied XPO1 mRNA expression with indicators of clinical outcome that help guide clinical decisions for management of metastatic ER^+^ tumors, e.g., PIK3CA, ESR1 and CCND1 expression. In both datasets, XPO1 mRNA level highly correlated with PIK3CA mRNA, supporting a potential XPO1 targeting in tumors with these clinically relevant genomic mutations found in 30−40% of ER^+^ metastatic tumors (Figure 1A). To examine pathways that might be affected by XPO1 and ER crosstalk in cells with ESR1 mutations, we performed a RNASeq experiment. To assess if single and combined treatments resulted in distinct gene expression patterns, we treated MCF7-ESR1^Y537S^ cells with 4-OHT, Fulv and Palb in the presence or absence of SEL. Unsupervised hierarchical clustering of differentially expressed genes showed that combining single treatments with SEL changed gene expression profiles (Figure 1B, Three clusters). To visualize if individual treatments resulted in distinct gene expression features, principle component analysis (PCA) was performed, which revealed about 65% of variation due to components 1, 2 and 3 (Figure 1B, Lower right panel). For all, co-treatment with SEL decreased genes associated with metastasis (Figure 1C). To test clinical utility of targeting XPO1 in endocrine resistant cells with high PIK3CA activity, we utilized a panel of cell lines with PIK3CA mutations (MCF7 cells and therapy-resistant derivatives) or high PIK3CA activity (BT474 cells) and tested combination of SEL with 4-OHT (active metabolite of tamoxifen), Fulv and Palb. In all the resistant cell lines tested, combining SEL with 4-OHT and Fulv resulted in synergistic reduction in cell viability compared to single agents (Figure 1D and Figure S1). SEL and Palb combination also resulted in reduced cell viability, even though the synergism was not observed in all of the cells line. (Figures S1 and S2).

Since our gene expression analysis showed that SEL synergized with currently used MBC therapies in clinic, we studied the impact of these combinations in various endocrine-responsive and -resistant BCa cell lines in hydrogels mimicking human tissue extracellular matrices from liver, bone and lung, the most common metastatic sites for ER^+^ tumors. To show that tissue-specific ECMs mimic human tissues and are a relevant for metastatic site-specific tumor models, we performed scanning electron microscopy (SEM) analysis, which showed that these ECMs have similar physical architecture and biophysical properties as the actual human tissues (Figure 1E,F). Since RNASeq analysis of MCF7-ESR1^Y537S^ cells grown on different tissue-specific ECM hydrogels showed that growth of cells on these matrices activated similar metastatic gene programs that were inhibited by SEL combination treatments as in Figure 1C, we performed colony formation assays. Combining 4-OHT, Fulv or Palb with SEL prevented colony formation of MCF7-ESR1^Y537S^ (Figure 1G,H) and T47D-ESR1^Y537S^, T47D-ESR1^D538G^, HCC1500, T47D-Parental and MCF7 parental cells grown on different tissue-specific extracellular matrix hydrogels (Figure S3).

### 2.2. Combination of SEL with Tamoxifen Inhibits Tumor Growth and Recurrence In Vivo

**Figure 2:**
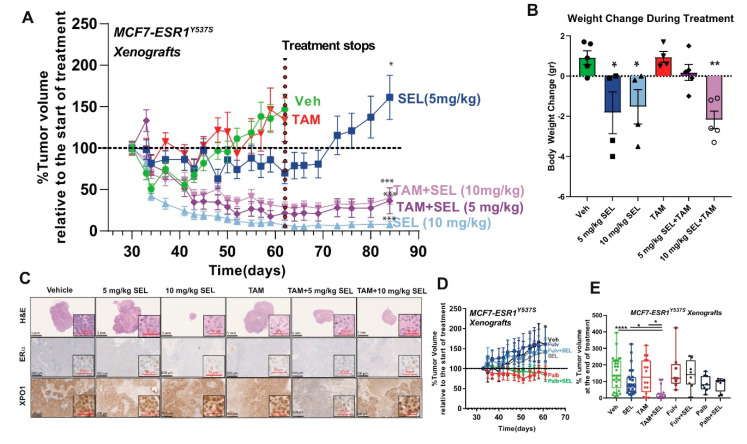
Combination of SEL with TAM inhibits growth of metastatic ER^+^ tumors in vivo. (**A**) SEL combination with TAM provides sustained tumor regression of MCF7-*ESR1^Y537S^* xenografts. A two-way ANOVA model was fitted to assess the time dependent contribution of ligand Vehicle (Veh), TAM) and inhibitor (Ctrl, 5 mg/kg SEL or 10 mg/kg SEL) treatment on tumor volume. When the main effects were statistically significant at *p* < 0.05, pairwise t-tests with a Bonferroni correction were employed to identify if treatment were statistically different from each other. * *p* < 0.05, ** *p* < *0*.01, *** *p* <0.001, **** *p* < 0.0001. (**B**) Percentage body weight change of mice. (**C**) Histological analysis of tumors from (A)**.** HE, ERα and XPO1 IHC stainings were performed. (**D**) Treatment with Fulv or Palb alone or in combination with SEL did not result in MCF7 *ESR1*^Y537S^ tumor regression. (**E**) Comparison of percent tumor volume change compared to start day of treatments in experiments from (A).

To validate our results in vivo, we generated xenograft tumors using MCF7-ESR1^Y537S^ cells. Tumor cells with ESR1^Y537S^ are differentially found in metastatic tumors and these models enabled us to test the utility of various SEL combinations in a model that mimics already metastasized tumors. TAM + SEL combination was very effective in reducing tumor size and maintaining tumor response even after the therapy was stopped (Figure 2A), with SEL at 5 mg/kg/mice, a concentration that does not affect animal weight (Figure 2B). The 10 mg/kg SEL and TAM + 10 mg/kg SEL treatment was more effective in terms of tumor regression, however, in these animals food intake and total body weight were lower. This response was similar to what we observed before in BT474 cells, tumors that initially regressed while on SEL only came back within 3 weeks after the treatment was stopped [11]. Histological analysis of the tumors showed that the tumors maintained ERα and XPO1 expression. Of note, XPO1 and ERα expression were lost in the only tumor that we could detect in the TAM + 5 mg/kg SEL group after we stopped treatment, explaining recurrence in this particular tumor (Figure 2C). Unexpectedly, Fulv or Palb combinations did not reduce tumor size at the concentrations used (Figure 2D,E). Overall, our results validated the feasibility of combining SEL with TAM to treat already metastasized ER^+^ tumors.

### 2.3. Single Agent Treatments and Growth on Metastatic Organ Sites Rewire Metabolic Pathways to Enhance Survival of BCa Cells

**Figure 3:**
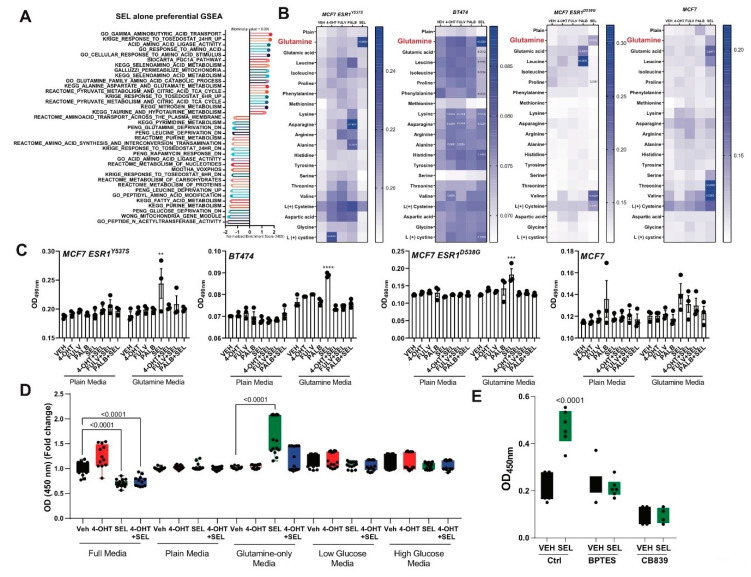
(**A**) Single agent treatments and growth on metastatic organ sites rewire metabolic pathways to enhance survival of BCa cells A. GSEA analysis showing activation of amino acid deprivation genes in MCF7-*ESR1^Y537S^* cells. (**B**) Endocrine-sensitive MCF7 cells, endocrine-resistant MCF7-*ESR1^Y537S^*, MCF7-*ESR1^D538G^* or BT474 cells were grown in minimal media containing only 2 mM individual amino acids. Cells were treated with different endocrine agents (1 µM 4-OHT, Fulv or Palb, and 100 nM SEL) and cell viability was quantified with WST-1 assay. All experiment conditions were repeated in six technical replicates and statistical significance values were calculated according to two-way ANOVA test. (**C**) Endocrine-sensitive MCF7 cells, endocrine-resistant MCF7-*ESR1^Y537S^*, MCF7-*ESR1^D538G^* or BT474 cells were grown in minimal media conditions supplemented only 2 mM individual amino acids. Cells were treated with different endocrine agents (1 µM 4-OHT, Fulv or Palb, in the presence or absence of 100 nM SEL) and cell viability of cells were quantified with WST-1 assay. All experiment conditions were repeated in six technical replicates and statistical significance values were calculated according to two-way ANOVA test. ** *p* < *0*.01, *** *p* <0.001, **** *p* < 0.0001. (**D**) Cell viability assay showing the effect of glutamine addition to the media. BT474 cells were cultured at a density of 2 × 10^3^ cells/well in a 96-well plate and treated with 1 µM 4-OHT alone and in combination with 100 nM SEL in different limiting media conditions. (**E**) Similar experimental conditions were tested in presence of 1 µM Glutaminase inhibitors, BPTES or CB839. A one-way analysis of variance (ANOVA) model was used for statistical significance of treatment and values were presented as mean ± SEM from three independent experimental repeats.

Since tumors that were treated with SEL did not maintain tumor regression, we further characterized this treatment to understand survival pathways that are activated in the presence of single agent treatments. To identify potential survival pathway that might be activated with SEL alone treatment, we compared gene sets that were differentially activated or repressed with SEL treatment. We found that SEL treatment resulted in regulation of several metabolic genes, which are related to amino acid metabolism and more specifically glutamine deprivation and metabolism programs (Figure 3A). To validate if this effect was due to glutamine with only SEL treatment, we grew various cell lines using media that contained only indicated amino acids. For all three endocrine resistant cell lines, MCF7-ESR1^Y537S^, MCF7-ESR1^D538G^ and BT474 cells, but not parental MCF7 cell, glutamine supplementation increased cell viability in the presence of SEL. Of note, different cell lines had increased cell viability with different single treatments potentially pointing out other mechanisms for tumor cell survival in the presence of different single agents (Figure 3B). Combining SEL with the various agents (4OHT, Fulv or Palb) resulted in inhibition of increased viability in minimal media with glutamine (Figure 3C). We validated the survival phenotype with SEL only with glutamine but not with low or high glucose or full media (Figure 3D). Glutamine only media also increased cell viability in MCF7-ESR1^Y537S^ cells (Figure S4A), as well as other conditions compared to Veh condition. Thus, to study the mechanism of SEL induced cell viability we focused on BT474 cells. In this cell line, glutamine- and SEL-increased cell viability was blocked by BPTES and CB836, showing dependence of this process on glutamine uptake and conversion into glutamate (Figure 3E).

**Figure 4:**
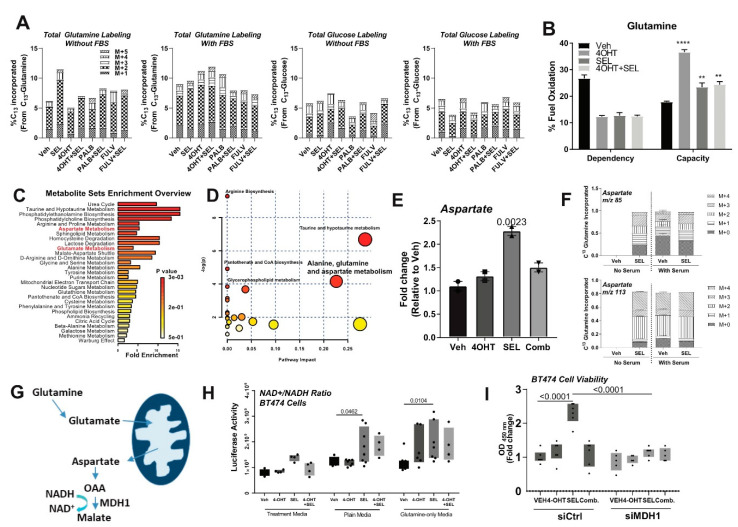
SEL only treatment induces glutamine-dependent aspartate synthesis to sustain NAD^+^ levels in the cell. (**A**) Total metabolite labelling after C13 glutamine or glucose flux in BT474 cells that were grown in the presence or absence of serum. (**B**) Mitochondrial fuel flex test in BT474 cells showing increased glutamine capacity in BT474 cells were cultured at a density of 3 × 10^4^/XFp plate wells, and treated with different endocrine agents (1 µM 4-OHT, Fulv and Palb) and in combination with 100 nM SEL. All results were visualized after normalizing OCR and ECAR values according to cell number. ** *p* < 0.01, **** *p* < 0.0001. (**C**) Metabolomics analysis result showing glutamine levels in SEL only treated BT474 cells. For this experiment, BT474 cells were cultured at a density of 2 × 10^5^ cells/10 cm plates in two technical repeats, and treated with different endocrine agents (1 µM 4-OHT, Fulv and Palb) and in combination with 100 nM SEL. (**D**) MetaboAnalyst pathway analysis. (**E**) Increased aspartate relative abundance identified by metabolomics analysis. (**F**) Increased C_13_ labelled-aspartate after C_13_ glutamine flux of BT474 cells. (**G**) Schematic representing pathway to replenish NAD+ from glutamine. (**H)** Detection of NAD^+^/NADH in the presence of defined media and different treatments. (**I**) Cell viability assay in the presence of glutamine only media with *MDH1* knock-down in BT474 cells.

Next, to study the fate of different fuels, glutamine or glucose, in the presence of complete or minimal media conditions (no serum added) and different treatments, we performed flux experiments using C_13_-labelled glutamine or glucose in BT474 cells. C_13_ incorporation into the cells increased in the presence of SEL when cells were grown in minimal media conditions with no serum. We did not replicate this effect with any of the other conditions (Figure 4A). We then monitored mitochondrial fuel dependency of the cells when we treated them with SEL only (Figure 4B). This analysis showed that there was an increase in dependence on glutamine, but not glucose or free fatty acids, for the maintenance of mitochondrial respiration, as evidenced by increased capacity in the presence of glutamine when the cells were treated with SEL (Figure 4B). Overall metabolomics analysis showed that metabolites that are in aspartate and glutamate metabolism pathways are among the most enriched metabolites with SEL only treatment (Figure 4C,D). Relative abundance of aspartate (Figure 4E) was significantly upregulated in the presence of SEL, and this increase was because of shunting of glutamine to aspartate synthesis as evidenced by increased C_13_ incorporation into aspartate (Figure 4F). Of note, we did not detect any aspartate labeling in Veh condition without serum. This pathway was previously reported to sustain NAD^+^ levels in cancer cells with mitochondrial defects (Figure 4G) [14,15,16]. To identify if supplementation with SEL increased production of metabolites, which were previously shown to be dependent on glutamine [17], we monitored NAD^+^/NADH ration, NADP^+^/NADPH ratio, reactive oxygen species and glutathione production (Figure 4H and Figure S4). We found that the increase in NAD^+^/NADH ratio was highest in cells that are grown in minimal media conditions supplemented with glutamine and SEL (Figure 4H and Figure S4B). In minimal media supplemented with glutamine, SEL-dependent cell viability was blocked by knock-down of MDH1 (Figure 4I), suggesting that glutamine is shunted to replenish NAD^+^ in a MDH1-dependent manner and increased cell survival under limited nutrient conditions or deregulated metabolic conditions in the tumor microenvironment.

### 2.4. PGC1α Drives the Metabolic Rewiring in Response to Metastatic Niche and Therapies

**Figure 5:**
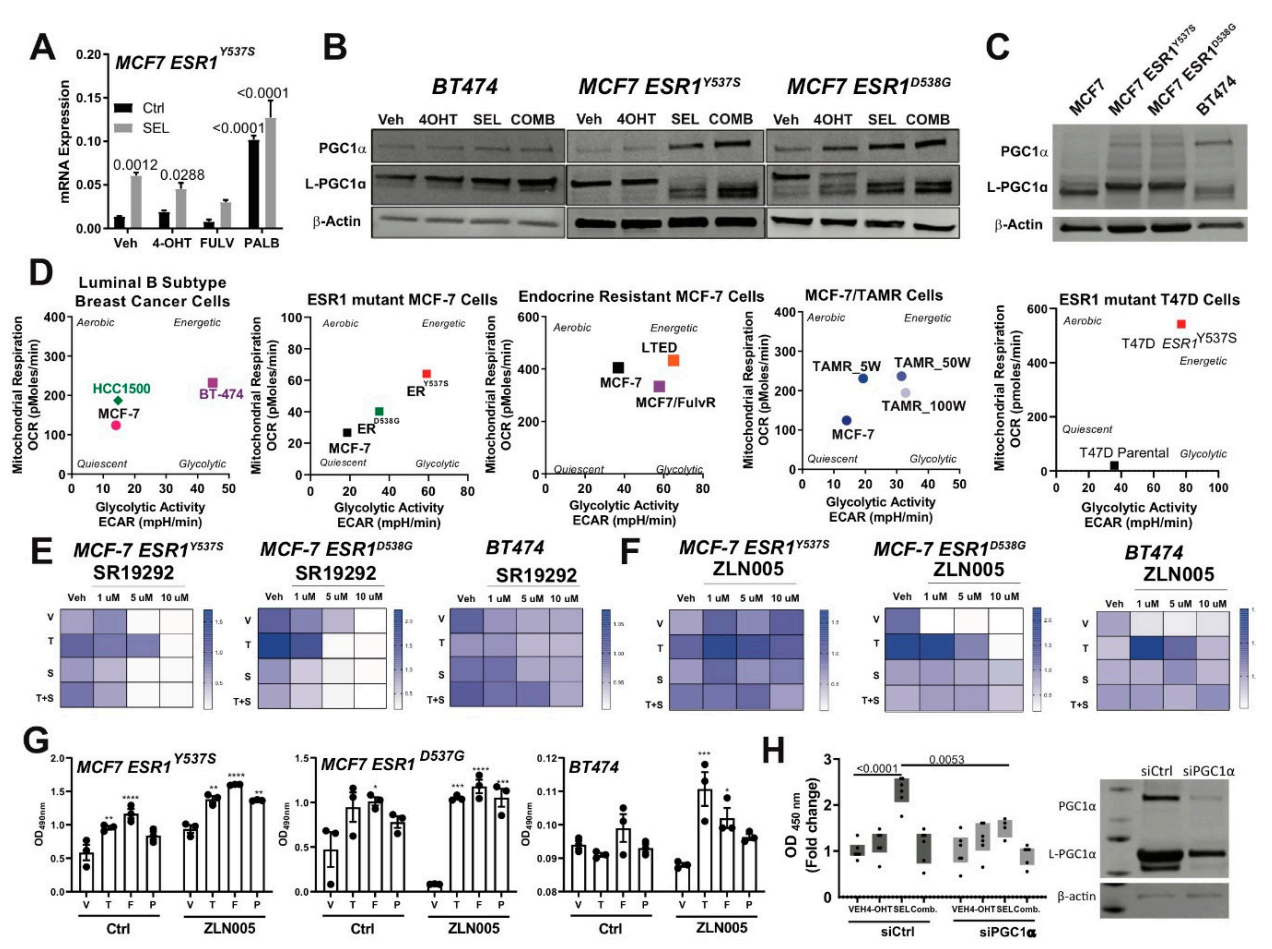
PGC1α drives the metabolic rewiring in response to metastatic niche and therapies. (**A**) RNA sequencing results showing SEL treatment induces *PPARGC1A (PGC1α)* mRNA expression alone or in combination with other therapy agents. Statistical analyses were analyzed according to one-way ANOVA test. Western blot analyses showing that PGC1α protein expression was relatively higher in cells that were treated with SEL (**B**) or in endocrine-resistant breast cancer cells (**C**)**.** All cell lines were seeded at a density of 2 × 10^5^ cells/30 mm plates. Cell lysates were prepared and western blot analysis was performed. (**D**) Cellular metabolic phenotype changes in different endocrine-sensitive and -resistant models were monitored with Seahorse metabolic profiling. Different de novo endocrine-resistant cell models (HCC1500, BT474, MCF7-*ESR1^D538G^* and MCF7-*ESR1^Y537S^*) and acquired resistant models (Tam^R^ cells generated with a consistent drug treatment strategy) were cultured at a density of 3 × 10^4^ cells/XFp plate wells, and treated with different endocrine agents (1 µM 4OHT, Fulv and Palb) and in combination with 100 nM SEL. The impact of PGC1α function on the cell viability of BT474 cells was tested. BT474 cells were seeded in 96-well plates, and treated with 1 µM 4-OHT alone and in combination with 100 nM SEL with a PGC1α inhibitor, SR19292 (**E**) or PGC1α activator, ZLN005 (**F**). A one-way analysis of variance (ANOVA) model was used for statistical significance of treatment and values were presented as mean ± SEM from three independent experimental repeats. Significances were compared according to first measurement values for each treatment condition. (**G**) The impact of PGC1α function on the cell viability of BT474 cells was tested. BT474 cells were seeded in 96-well plates, and treated with 1 µM 4OHT, Fulv or Palb alone and in combination with 100 nM SEL with ZLN005. * *p* < 0.05, ** *p* < *0*.01, *** *p* <0.001, **** *p* < 0.0001. (**H**) Cell viability assay in the presence of glutamine only media with PGC1α knock-down in BT474 cells.

Our RNA-Seq analysis showed that PPARGC1A (PGC1α) mRNA [13], (Figure 5A) was induced by SEL treatment in endocrine resistant cell lines and protein expression of PGC1α was also increased (Figure 5B). PGC-1α is a major regulator of mitochondrial biogenesis, and overexpression of this protein was implicated in regulation of glutamine metabolism [18] and resistance to metabolic drugs [19]. Therefore, we hypothesized that this protein might be involved in the metabolic rewiring and different outcomes we observed with different treatments. Endocrine resistant cell lines had higher PGC1α expression compared to parental cells (Figure 5C), consistent with higher mitochondrial and glycolytic respiration in these cell lines compared to endocrine sensitive cell lines (Figure 5D). Small molecule inhibitor of PGC1α dose-dependently inhibited therapy-resistant cell growth (Figure 5E and Figure S5A,B). Small molecule activator of PGC1α, ZLN005 increased therapy resistant cell line viability with 4-OHT (T) at low dose, which was blocked by SEL combination (S) (Figure 5F). At this dose, ZLN005 also potentiated agonistic activity of Fulv (F) and Palb (P) in different therapy resistant cell lines (Figure 5G and Figure S5A,B). Knockdown of PGC1α prevented SEL-induced growth of BT474 cells in glutamine containing media (Figure 5H).

**Figure 6:**
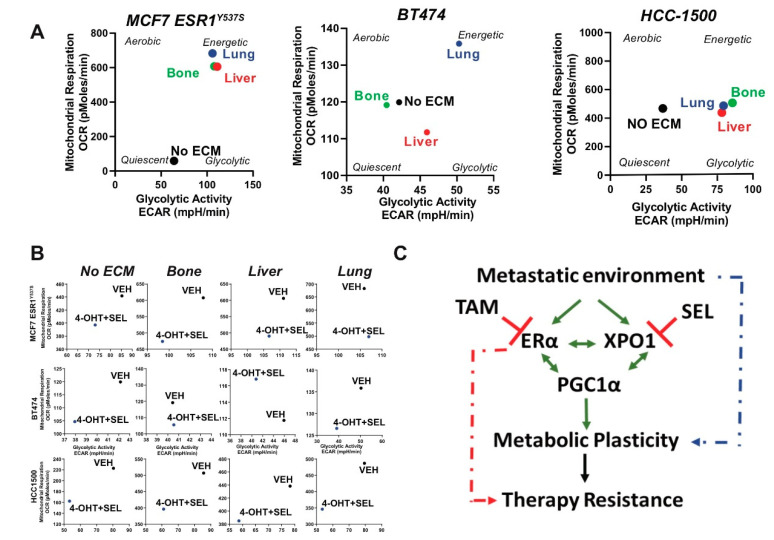
(**A**) Cellular metabolic phenotypes of MCF7-*ESR1^Y537S^* cells were determined by seeding 3 × 10^4^ cells/XFp plate well in different tissue-specific extracellular matrix environments. Cellular metabolic phenotypes of MCF7-*ESR1*^Y537S^, BT474 and HCC1500 cells were determined by seeding 3 × 10^4^ cells/XFp plate well in different tissue-specific extracellular matrix environments with SR-18292. All Seahorse assays were repeated two times in three technical replicates. OCR and ECAR values were normalized according to cell number. (**B**) Cellular metabolic phenotypes of MCF7-*ESR1^Y537S^*, HCC1500, or BT474 cells were determined by seeding 3 × 10^4^ cells/XFp plate well in different tissue-specific extracellular matrix environments with 4-OHT + SEL combination. All Seahorse assays were repeated twice with three technical replicates. OCR and ECAR values were normalized according to cell number. (**C**) Schematics of how TAM + SEL combination works to prevent metabolic plasticity and therapy resistance.

Growing MCF7-ESR1^Y537S^ cells and other endocrine resistant cell lines on an extracellular matrix from the bone, liver or lung tissue (major sites for BCa metastasis) resulted in an increase in both glycolytic and mitochondrial activity, suggesting metastatic site-specific metabolic rewiring taking place when cells are adapting to new microenvironments (Figure 6A). Treatment of cells with 4-OHT + SEL combination resulted in a reduction in metabolic energetics of the cells (Figure 6B). RNA-Seq analysis also showed that when we combined 4-OHT and SEL the expression of targets of PGC1α was reduced (Figure S5C). Our results showed that treatment with single agents, or growth in tissue-specific ECM hydrogels mimicking metastatic tissues affected metabolic phenotype of BCa cells and inhibiting PGC1α or co-targeting ERα and XPO1 reduced this effect of extracellular matrix. This data emphasizes the therapeutic potential of these combinations in reducing metastatic tumor growth and adaptation to stress conditions and preventing therapy resistance in already metastasized tumors (Figure 6C).

## 3. Discussion

In this study, we elucidated the causes and mechanisms of therapy resistance by focusing on the role of metabolic changes in tumor cells enabling them to adapt to and survive in metastatic niches. We tested therapeutic potential of combining XPO1 inhibitors with currently utilized clinical drugs to prevent therapy resistance during metastatic tumor treatment. We found that single therapies activated multiple metabolic adaptations, one of which involved utilization of glutamine to replenish NAD^+^. Our analysis identified PGC1α dependent metabolic changes as a mechanism to adapt to metastatic sites and reduce the effectiveness of therapies.

Our studies established XPO1 as a target, whose inhibition would enhance the effectiveness of current therapies, by preventing metabolic adaptation and improved survival in metastatic organ sites during sequential therapies. Resistance to therapies is a significant concern for metastatic breast cancer patients. The majority of current research efforts in the therapy resistance field are focused on a delineation of the underlying mechanisms that lead to increased activity of selective signaling pathways. Undoubtedly, interrogating and targeting the end-point kinases in tumors is highly relevant and these studies led to the development of combination therapies involving PI3K inhibitors or mTOR pathway inhibitors together with endocrine agents. However, resistance to these combination therapies also occurs, and in such cases, the cancer that develops is considerably more aggressive due to hyperactivation of compensatory mitogenic signaling pathways [20]. Moreover, these kinase inhibitors have many adverse side effects. Additionally, *ESR1* mutations that decrease sensitivity of the receptor to endocrine therapies were identified in about 15–40% of the metastatic, but not primary tumors. *PIK3CA* mutations are found in about 30−40% of tumors. Our proposed approach might offer an alternative treatment strategy to prevent emergence of these adaptive strategies for cancer cells and therapy resistance, which is a significant clinical challenge currently.

We used tissue-specific ECM hydrogels to characterize MBC cell phenotypes and responses to drugs. These hydrogels are obtained from decellularized porcine tissue, and composition is characterized by mass spectrometry and quantitative biochemical and biophysical assays and have been shown to contain tissue characteristic extracellular matrix proteins as well as growth factors [21,22]. Scanning electron microscopy analysis of decellularized tissues revealed structural similarity and rheometry analysis showed similarities in the biophysical properties of the hydrogels to actual human tissues. Stiffness is a critical regulator of cancer cell phenotype [23]. A key consideration about the mechanical stiffness, i.e., resistance to deformation, of cell culture substrates is accounting for the enormously broad range of stiffness that is commonly applicable to in-vitro cell culture systems. Substrate stiffness typically range from quite stiff (e.g., tissue culture plastic/polystyrene: approximately 1GPa) to quite soft (e.g., hydrogels: approximately 1–10 kPa). The stiffness of most soft, healthy tissues in the human body, including liver and lung, range from 1–10 kPa [24]. The stiffness of tissue culture plastic and glass range from 1-3GPa-approximately six orders of magnitude, or one million times, stiffer than soft tissues in the human body. Notably, the modulus values of our liver and lung ECM hydrogels are within a physiologically-relevant range, especially compared to plastic. Thus, hydrogels provide an opportunity to reconstitute metastatic site environment and study drug responses and metastasis-associated phenotypes in vitro.

Tumor cells can create favorable environments in metastatic tissues by secreting various factors even before they metastasize [25]. These pre-metastatic niches (PMNs) are characterized by altered local deposition of extracellular matrices, a rudimentary vascular network, recruitment of bone marrow derived cells (BMDCs) and presence of proinflammatory molecules and cells [26]. There is a current shortfall of relevant in vitro models that accurately reflect the PMNs for ER^+^ tumors, as well as novel analysis tools to understand the molecular mechanisms driving adaptation and survival of metastatic ER+ breast cancer cells in these environments. Therefore, using tissue-specific extracellular matrices to mimic metastatic tissue sites for ER^+^ tumors enabled us to study the impact of PMN microenvironment on how ER^+^ BCa cells behave, adapt, and respond to therapy agents in metastatic tissue environments. Future studies are needed, which expand these models by addition of other cell types (CAFs, BMDCs and immune cells) and monitoring responses to therapies.

Cancer cell metabolism plasticity provides a novel route for therapy resistance [27]. NAD^+^ is a critical metabolite that links cellular metabolism, signaling and transcription [28]. In addition to providing ATP, TCA cycle and glycolysis regenerates NAD^+^ levels. When glycolysis is inhibited, glutamine, through conversion to glutamate and α-Ketoglutarate, is utilized by the TCA cycle to produce ATP and regenerate NAD+. When glycolysis and TCA cycle is blocked, glutamine is converted to aspartate through a series of steps that regenerates NAD^+^ by cytosolic MDH1 [14,15,16]. Activation of this mechanism explained why tumors treated with SEL alone come back in vivo and further emphasized the importance of combining TAM and SEL to treat tumors and prevent further recurrence for metastatic tumors. Our data showed that in minimal media containing glutamine, single agent SEL treatment actually increased cell viability through Gln metabolism via aspartate conversion, as opposed to causing cancer death as is observed under full media conditions. Since tumors have metabolic heterogeneity, we speculate that recurrences from tumors treated with single agent SEL could result from cellular populations of the original tumor with limited amino acid content, which relied on glutamine metabolism and were therefore not susceptible to SEL-induced cell death.

Our research represents a new and substantive departure from the status quo by shifting the focus to inhibiting dynamic mechanisms enabling adaptation to metastatic tissue environments and therapies. PGC1α was previously implicated in metabolic rewiring and drug resistance of triple negative BCa, yet the role of PGC1α has been controversial [19,29,30,31]. PGC1α is involved in the progression of primary hormone-related cancers by physically interacting with, and acting as a coactivator of ERα [32,33]. Additionally, local estrogen biosynthesis is promoted by TNFα induced PGC1α activity in endometrial cancer cells [34]. The synergism between ERα and PGC1α activates anti-apoptotic pathways in breast and ovarian cancer cells by manipulating mitochondrial membrane integrity [35]. Our observation that activation of PGC1α promote survival of metastatic ER^+^ breast cancer cells and adaptation mechanisms is novel. In addition, we observe changes in different isoforms of PGC1α (full length and L-PGC1α), which might have different roles. Future studies are needed to dissect the contribution of each isoform to therapy resistance and metastatic site-specific metabolic adaptation mechanisms in advanced breast cancers as well as in other cancer types.

## 4. Materials and Methods

### 4.1. Cell Culture, Ligand Treatments

All cell lines were obtained from American Type Culture Collection (Manassas, VA). MCF7 (ATCC^®^ HTB-22^TM^) (RRID:CVCL_0031) and T47D (ATCC^®^ HTB-133^TM^) (RRID:CVCL_0553) parental cells were cultured in Roswell Park Memorial Institute (RPMI-1640) with NEAA salts (Sigma, St. Louis, MO, USA), 5% fetal bovine serum (FBS) (HyClone, Logan, UT), 100 µg/mL penicillin/streptomycin (Invitrogen, Carlsbad, CA, USA) and 50 mg/mL Gentamicin (Gibco, Gaitersburg, MD, USA). MCF7/*ESR1^D537S^* and -*ESR1^Y537S^* cells (RRID:CVCL_0031) were generated as described in [36] and were cultured in Dulbecco’s Modified Eagle Medium (DMEM) with NEAA salts, 10% FBS, 100 µg/mL penicillin/streptomycin and 50 mg/mL Gentamicin. T47D *ESR1^D537S^* and T47D *ESR1^Y537S^* cells (RRID: CVCL_0553) were cultured in Modified Eagle Medium (MEM) with NEAA salts, 10% FBS, 100 µg/mL penicillin/streptomycin and 50 mg/mL Gentamicin. Human breast cancer cell lines, BT474 (RRID: CVCL_0179), HCC1500 (RRID:CVCL_1254) and MDA-MB-134 (RRID:CVCL_0617), were used as a model of de novo resistance [11,37,38]. BT474 were cultured in ATCC recommended Hybri-care medium with 10% inactivated FBS, sodium bicarbonate and antibiotics. MDA-MB-134 cells were cultured in Leibovitz’s medium with 20% FBS, and 100 g/mL penicillin/streptomycin (Invitrogen). HCC1500 cells were cultured in ATCC-formulated RPMI-1640 media with 10% FBS, sodium bicarbonate and antibiotics. Acquired resistance was studied from resistance progression cell lines previously derived and characterized by long-term exposure of parental MCF7 cells to 4-OHT (Tam^R^) [11,37]. These cells retain ERα expression, do not require E2 for growth, are not growth inhibited by SERMs, and 4-OHT stimulates their growth [11,37]. Cells were authenticated by checking activity and expression of ERα, and proliferative responses to various drugs used in this study for all cell lines, and by sequencing of MCF7/*ESR1*^Y537S^ and MCF7/*ESR1*^D537G^ cells as described.

To generate drug resistant breast cancer cell lines, parental cells were treated with 10^−7^ M 4-OHT, Fulvestrant (Fulv) and/or Palbociclib (Palb) for 25 weeks. Drug treatments were repeated twice weekly (Monday and Friday). Resistant colonies were selected and stored in liquid nitrogen every five weeks.

For drug treatments, cells were incubated with media containing 10^−6^ M 4-OHT (Sigma), 10^−6^ M Fulv (Sigma), 10^−6^ M Palb (Sigma) alone or in combination with 10^−7^ M selinexor (SEL) (Karyopharm Therapeutics, Boston, MA, USA) for 24 h. For other drug treatments, SR-18292 (PGC1α inhibitor) (Selleckchem) and ZLN005 (PGC1α activator) (Selleckchem) were used at concentrations between 1 × 10^−5^ M, 5 × 10^−6^ M and 1 × 10^−6^ M. MK2206 (PI3K inhibitor) was used at a concentration of 5 × 10^−5^ M [36,39,40,41].

### 4.2. Cell Viability Assays and siRNA Knock Down

Cells were seeded at a density of 2 × 10^3^ cells/well in a 96-well plate, and were grown overnight in corresponding media with/without phenol red. On day 1, cells were treated with drugs alone and in combination. Treatments were repeated on day 4. On day 7, the effect of different drug treatments on the cell viability was assayed by using WST-1 reagent. Results were quantified by Cytation5 plate reader by measuring absorbance at 450 nm (BioTek, Winooski, VT, USA). Statistical analyses were done by using Graphpad© Prism8 software (GraphPad Software Inc., La Jolla, CA, USA, www.graphpad.com). All experiment conditions had six technical repeats and experiments were repeated at least for three times. WST1 assay was performed to quantify cell viability.

For siRNA knock down experiments, cells were seeded at a concentration of 2 × 10^3^ cells/well in corresponding treatment media without phenol red and antibiotics. Next day, cells were incubated with 25 nM siRNAs targeting *PPARGC1a* (Dharmacon, ON-TARGET plus SMARTpool Human *PPARGC1a* (10891)) and *MDH1* (Dharmacon, ON-TARGET plus SMARTpool Human *MDH1* (4190)) genes. Target sequences of both siRNAs were given as below (J-005111-05, PPARGC1a: GAGAAUUCAUGGAGCAAUA), (J-005111-06, PPARGC1a: GAAGAGCGCCGUGUGAUUU), (J-005111-07, PPARGC1a: ACACUCAGCUAAGUUAUAA), (J-005111-08, PPARGC1a: GCAGGUAACAUGUUCCCUA), (J-009264-09, MDH1: CCUUAGAUAAAUACGCCAA), (J-009264-10, MDH1: GGGAGAAUUUGUCACGACU), (J-009264-11, MDH1: CAACAGAUAAAGAAGACGU), (J-009264-12, MDH1: AGGUUAUUGUUGUGGGUAA). Cells were incubated with the media containing siRNAs for 48 h. Cells were incubated with the media containing siRNAs for 48 h. Cells were treated with single agents or in combination with SEL twice on days three and six. On day nine, cell viability was measured and analyzed as explained above.

For minimal media experiments, MCF7, MCF7-*ESR1^Y537S^*, MCF7-*ESR1^D538G^*, and BT474 cells were seeded at a density of 2 × 10^3^ cells/well in 96 well plates. Next day, cells were treated with 10^−6^ M 4-OHT, Fulv or Palb and 10^−7^ M SEL alone and in combination (Plain media: DMEM containing only 7.5% sodium bicarbonate, Low glucose media: DMEM + 1 g/L D-Glucose, High glucose media: DMEM+4.5 g/L D-Glucose, Glutamine-only media: DMEM + 2 mM L-Glutamine). Treatments were repeated on day 4. On day 7, cell viability was measured using WST-1 reagent.

For minimal media with individual amino acid experiments, parental MCF7, MCF7-*ESR1^Y537S^*, MCF7-*ESR1^D538G^*, and BT474 cells were seeded at a density of 2 × 10^3^ cells/well in 96 well plates. On day 1, cells were treated with DMEM media containing 2 mM individual amino acids. Amino acid treatments were repeated on day 4. On day 7, cell viability was measured using WST-1 reagent.

### 4.3. 3-D Cell Culture Models

IN SITE^TM^ Metastasis Kit (Xylyx Bio, Inc., NY, USA) containing TissueSpec^®^ Bone (MTSBN101), Liver (MTSLV101) and Lung (MTSLG101) ECM Hydrogels), was used to model tumor microenvironments according to manufacturer protocol. Briefly, 2 × 10^3^ cells were encapsulated in corresponding tissue-specific ECM hydrogels by mixing them with tissue culture matrix. A mixture volume of 100 uL/well was placed in 96-well plates in triplicates. Plates were incubated at 37 °C in a humidified environment with 5% CO_2_ for at least 45 min to achieve gelation. Cells were treated with media containing different drugs alone or in combination with SEL every Monday and Friday for three weeks. Oncosphere formations were visualized by Invitrogen^TM^ EVOS^TM^ XL Core Light Microscope (4× and 25× magnifications) (Waltham, MA, USA). OpenCFU colony counting software (http://opencfu.sourceforge.net/) was used to automatically count colony number and size. All statistical analyses were completed by using GraphPad© Prism8 software (RRID:SCR_002798, San Diego, CA, USA).

### 4.4. Scanning Electron Microscopy

Bone, liver and lung tissue and matrix decellularized tissue (extracellular matrix) samples were collected, fixed in formalin for 24 h, rinsed in 70% ethanol, frozen, lyophilized, and imaged using an electron microscope (Gemini SEM 300, Zeiss, Oberkochen, Germany) with accelerating voltage 2.5 kV.

### 4.5. Mechanical Characterization of IN SITE^TM^ Metastasis ECM Hydrogels

Stiffnesses of TissueSpec^®^ Bone, Liver, and Lung ECM Hydrogels (#MTSMS10, IN SITE^TM^ Metastasis ECM Hydrogel Kit, Xylyx Bio, Brooklyn, NY, USA) were determined by rheometry analysis. ECM hydrogels (6 mg/mL) were prepared in cylindrical molds to obtain hydrogel disks (volume: 1 mL, diameter: 13 mm), which were transferred onto the Peltier plate of a shear strain-controlled rheometer (DHR-2, TA instruments, New Castle, DE, USA). Hydrogels were evaluated in a parallel plate geometry using a plate with a sand-blasted surface to secure hydrogels and prevent slipping. After strain sweeps (0.1–30%) were performed to determine linear viscoelastic regions of hydrogels, oscillatory frequency sweeps (0.1–10 s^−1^) were performed with constant shear strain (5%) to assess the viscoelastic properties of hydrogels. Storage modulus (G’) was plotted against frequency to determine stiffness (elastic response). All hydrogels were tested in triplicate, and values represent mean ± standard errors.

### 4.6. In Vivo Xenograft Study, Immunohistochemistry Staining (IHC) and Data Analysis

All experiments involving animals were conducted with protocols approved by the University of Illinois at Urbana-Champaign and following the National Institutes of Health standards for use and care of animals (IACUC Protocol 14193). Tumor xenograft studies were performed using the MCF7 *ESR1^Y537S^* cells. Briefly, we used 6-week-old BALB/c athymic (RRID: IMSR_TAC:balb), ovariectomized, nude female mice from Taconic Biosciences. Power calculations were done based on our previously published studies and five animals per group were used [11,13]. After one week of acclimatization, we injected 1 × 10^6^ cells resuspended in 50% PBS and 50% Matrigel subcutaneously into both the right and left flank of each mouse. Once the tumor size reached 200 mm^3^, we randomized five animals to each treatment group. Half of the mice were implanted with vehicle pellets and the other half were implanted with 25 mg; 60-day release TAM pellets (Innovative Research of America). We then randomized each group to Veh or SEL (5 mg/kg, 5 times per week; or 10 mg/kg 3 times per week). SEL is administered orally for 4 weeks. Animals were injected with Fulv (5 mg/kg, intramuscular injection) once weekly [42], or PALB (100 mg/kg) was delivered by oral gavage twice weekly [43]. SEL concentrations were selected based on clinically relevant dose [44,45,46]. Animals were monitored daily by the veterinarians for any signs of dehydration, stress, and pain. Total weight, food intake, and tumor size were measured by blinded researchers using a digital caliper biweekly. Tumor volume was calculated by length × width^2^/2.

Tumors were removed from euthanized mice at the end of the experiment or at the time when tumor size reached 1000 mm^3^ and were fixed in 10% neutral-buffered formalin, processed, and embedded in paraffin in 2M sucrose before being frozen in cutting medium. Tissues were cut in 5-micron sections by using a microtome (The Leica RM1255, Austria). For both XPO1 and ERα immunostainings, tissues were deparaffinized and hydrated through graded alcohols to water. Antigen retrieval was performed by using citrate buffer, pH 6.0 in a steamer for 1 h. Samples were blocked in hydrogen peroxide for 10 min. To remove non-specific protein staining, samples were blocked with Background buster (Innovex Biosciences, Richmond, CA, USA) for 10 min and rinsed with TBS-Tween solution, pH 7.6. Then, samples were incubated with XPO1 (Bethyl laboratories, A300-469A) or ERα (MC-20, Santa Cruz Biotech) (RRID: AB_631470) primary antibody overnight at 4 °C. After rinsing with TBS-Tween solution, pH 7.6, samples were stained with secondary anti-rabbit and anti-mouse HRP-Polymer (Biocare Medical, Concord, CA) for 30 min. Finally, samples were incubated with DAB (Innovex, Richmond, CA) for 5 min and counterstained with hematoxylin, dehydrated and mounted on slides. Visualization of the samples were performed with Nanozoomer Slide Scanner (Hamamatsu, Japan) at 80× magnification and positive staining quantification was performed by NDP software.

### 4.7. Western Blot Analysis in Cell Lines

Cells were seeded on 30 mm cell culture plates at 1 × 10^5^ cells/well in corresponding media and were treated with media containing drugs alone or in combination with SEL. Next day, cells were collected in lysis buffer (0.5 M EDTA, 1 M TrisHCl pH 8.1, 10% SDS, 10% Empigen, ddH2O) with 1X Complete Protease Inhibitor (Roche) and 1X Phosphatase Inhibitor (Thermo Scientific, Waltham, MA, USA). Cell lysates were sonicated and protein concentrations were determined by BCA assay (Thermo Scientific). Samples were boiled in SDS-containing loading buffer and 10-20 µg of each sample was run in 10% precast gels (BioRad) and transferred to nitrocellulose membrane. The membranes were blocked in Blocking Buffer (Odyssey^®^, Li-Cor, Lincoln, NE, USA) and target proteins were probed with XPO1 (RRID:AB_2215815) (sc-74454, Santa Cruz Biotechnology, Dallas, TX, USA), p-Akt S473 (#4060, Cell Signaling) (RRID:AB_2811246), pAkt T308 (#13038, Cell Signaling) (RRID:AB_2629447), Akt (#9272, Cell Signaling) (RRID:AB_329827), PGC1α (#SAB2500781, Sigma-Aldrich) (RRID:AB_10604141) antibodies in 1:1000 dilution and β-actin (Sigma SAB1305546) (RRID:AB_2541177) antibody in 1:10000 dilution. The secondary antibodies obtained from Odyssey were used at 1:10000 dilution. The membranes were visualized by using Licor Odyssey CLx infrared imaging device and software. All results were repeated two times and the results were normalized to total protein or β-actin loading control. Representative blots are presented.

### 4.8. Gene Expression Analysis and XPO1 and PIK3CA Expression Analysis Using TGCA and METABRIC Data

RNASeq analysis was performed as previously described [18,19,20]. Briefly, BT474 cells were treated with Vehicle (Veh, 0.5% EtOH), 10^−6^ M 4OHT (Sigma), 10^−6^ M Fulv (Sigma), 10^−6^ M Palb (Sigma) alone or in combination with 10^−7^ M SEL (Karyopharm) for 24 h. Concentrations of ligands are based on our previously published study [11] and clinical data [45,46]. Total RNA was extracted with TRIzol reagent (Life Technologies, Carlsbad, CA, USA) according to the manufacturer’s protocol and cleaned using a clean-up kit (QIAGEN, Hilden, Germany). RNA quality was assessed using bioanalyzer. Total RNA from each sample (three per treatment group) were sequenced at the UIUC sequencing center, and data was generated in Fastqc file format to compare the expressions between the four treatment groups. Preprocessing and Quality Control: Fastqc files containing raw RNA sequencing data were trimmed using Trimmomatic (Version 0.38) (RRID: SCR_011848) [47]. Next, the reads were mapped to the Homo sapiens reference genome (GRCh37) from the Ensembl (RRID:SCR_002344) [48] database and aligned using the STAR alignment tool (RRID: SCR_015899) (Version 2.7.0f) [49]. After this, the read counts were generated from SUBREAD (Version 1.6.3) (RRID:SCR_009803) [50] and featured counts were exported for statistical analysis in R. Quality control and normalization was conducted in R using edgeR (Version 3.24.3) (RRID:SCR_012802) [51]. Raw data is available from GEO (GSE136823).

### 4.9. Statistical Analysis and DEGs

Statistical analysis was conducted in R using limma (Version 3.38.3) (RRID:SCR_010943) [52,53]. Empirical Bayesian statistics were conducted on the fitted model of the contrast matrix. Differentially expressed genes were then determined by fold-change and *p*-value with Benjamini and Hochberg multiple test correction for each gene, for each treatment relative to the vehicle control. We considered genes with fold-change >1.5 and *p*-value < 0.05 as statistically significant, differentially expressed. Cluster3 software was used for clustering the differentially expressed genes. Data was visualized using Treeview Java. PCA analysis was performed using StrandNGS (Version 3.1.1, Bangalore, India). GSEA [54,55] analysis was used to identify GO terms associated with different treatments.

cBioPortal for Cancer Genomics (https://www.cbioportal.org/) (RRID:SCR_014555) website was used to assess the correlation between XPO1 expression and expression of other most commonly mutated genes in ER^+^ tumors. To identify co-expression profiles of *XPO1* and *PIK3CA* in different breast tumors, METABRIC [56] and TCGA [57] data were selected. Selection criteria was narrowed down to ER^+^ tumors. Then, expression of *PIK3CA* and *XPO1* was plotted in ER^+^ tumors [58,59].

To visualize and reduce the dimension of gene expression features principle component analysis (PCA) was performed. The original dataset, with dimensions of about 7000 by 24, was transposed so that we could calculate principle components by treatment (VEH, SEL, 4OHT, PAL, FUL, 4OHT + SEL, PAL +SEL, FUL + SEL). Gene expression features were unit scaled with a mean equal to 0 and a standard deviation equal to one. A decomposition algorithm was applied to the gene expression matrix and PCs were calculated. The code is provided in https://github.com/brandis2/breast_cancer_combined_targeting/blob/master/3-D%20PCA%20analysis.md.

### 4.10. Metabolomics Analysis

Metabolomics analysis was performed as described [60]. Briefly, BT474 and MCF7-*ESR1^Y537S^* cells were seeded at a density of 2 × 10^5^ cells/10 cm plate in treatment media. Next day, they were treated with 10^−6^ M 4OHT and 10^−7^ M SEL alone or in combination for 24 h. Metabolites were extracted using acetonitrile/methanol/water and samples were submitted to the Metabolomics Center at UIUC. GC/MS whole metabolite profiling was performed to detect and quantify the metabolites by using Gas chromatography-mass spectrometry (GC/MS) analysis. The hentriacontanoic acid was added to each sample as the internal standard prior to derivatization. Metabolite profiles were acquired using an Agilent GC-MS system (Agilent 7890 gas chromatograph, an Agilent 5975 MSD, and an HP 7683B autosampler, Lexington, MA, USA). The spectra of all chromatogram peaks were evaluated using the AMDIS 2.71 and a custom-built database with 460 unique metabolites. All known artificial peaks were identified and removed prior data mining. To allow comparison between samples, all data were normalized to the internal standard in each chromatogram. Metabolomics data with sample class annotations (Veh, 4-OHT, SEL, 4-OHT + SEL) were uploaded to the Statistical Analysis tool of MetaboAnalyst software version 4.0 (RRID:SCR_015539) [61]. Data were log transformed and scaled using Auto scaling feature. VIP scores for top 25 metabolites that discriminate between different treatment groups were calculated and displayed using the Partial Least Squares-Discriminant analysis tool. Heatmap of class averages of 25 metabolites was generated using Heatmap feature using default options for clustering and restricting the data to top 25 metabolites ranked by t-test. Enrichment analysis and pathway analysis tools were used to identify metabolic pathways associated with enriched metabolites.

### 4.11. Flux Analyses

BT474, MCF7 *ESR1^D537S^* and MCF7 *ESR1^Y537S^* cells were seeded at a density of 2 × 10^5^ cells in 10 cm plate in corresponding growth media containing 10% FBS, 11 mM or 25 mM D-Glucose and 2 mM L-Glutamine. Next day, media was replaced with media containing only 10% dialyzed FBS (all amino acids were removed via dialyses) and 2 mM isotope labeled [U-13C5, 99%] L-Glutamine (Cambridge Isotope Laboratories, MA, USA). Cells were incubated with this media for 24 h and then cells were collected in Acetonitrile (Sigma, MO, USA) and kept at −80 °C until they were submitted to the Roy J. Biotechnology Center Metabolomics Facility for GC/MS analyses. NTFD software was used to analyze data. Average from three replicates and 95% confidence intervals for the relative mass isotopomer abundances were plotted.

### 4.12. Seahorse Metabolic Profiling Assays

MCF7, MCF7 Tam^R^ and BT474 cells were seeded at a density of 5 × 10^4^ cells/well of 8-well Seahorse plates. MDA-MB-134 and HCC1500 cells were seeded at a density of 1 × 10^5^. MCF7 *ESR1^D537S^*, MCF7 *ESR1^Y537S^*, T47D *ESR1^Y537S^* and T47D *ESR1^D537S^* cells were seeded at a density of 3 × 10^4^ in corresponding treatment media without phenol red in each well of the XFp Cell Culture miniplates, respectively (Seahorse Bioscience Inc., Billerica, MA, USA). The FCCP concentration was 0.5 µM for MCF7, BT474, MDA-MB-134 and HCC 1500 cells. Next day, cells were treated with treatments media containing endocrine agents alone or in combination with SEL overnight and the cartridges were hydrated with the calibration solution and kept in a non-CO_2_ incubator at 37 °C overnight. In parallel, a duplicate of each plate was used for cell counting to monitor cell number changes after 24 h of treatments and Seahorse data was normalized to total cell number. On the assay day, cells were washed with XF Base Media without phenol red (Seahorse Bioscience Inc., Santa Clara, CA, USA) supplemented with 10 mM L-glucose, 2 mM L-glutamine (Gibco) and 1 mM sodium pyruvate (Gibco, Waltham, MA). The ECAR (mpH/min) and OCR (pmol/min) values were obtained by using Seahorse XFp Cell Energy Phenotype Test Kit (Seahorse Bioscience Inc.), which were run with Seahorse XFp Analyzer (Seahorse Bioscience Inc.). Experiments were performed in triplicate and repeated at least three times.

For the Seahorse experiments conducted in different tissue-specific extracellular matrix conditions, Seahorse XFp plates were coated with different Native Coat^TM^ ECMs for bone, lung and liver (XYLYX, Brooklyn, NY, USA). Then, these 2D coats were incubated at 37 °C in a humidified environment with 5% CO_2_ for at least 1 h. Cells were seeded in these coated Seahorse plates after removing native coat mixtures. The rest of the treatment and assay processes were applied as explained above.

### 4.13. Luminescence-Based Assays

NAD^+^/NADH-Glo^TM^ Assay (Promega, Madison, WI), NADP^+^/NADPH-Glo^TM^ Assay (Promega, Madison, WI), Reactive oxygen species (ROS)-Glo^TM^ (Promega, Madison, WI, USA), Glutathione (GSH)–Glo^TM^ Assay (Promega, Madison, WI, USA) were run based on same procedure. BT474, MCF7 *ESR1^Y537S^* and T47D *ESR1^Y537S^* cells were seeded at a concentration of 2 × 10^3^ cells/well in 96-well plates in corresponding phenol red-free media. Next day, cells were treated with endocrine agents alone or in combination with SEL in corresponding concentrations, and they were incubated with drugs for 24 h. All assays were run according to the supplier’s instructions for use of products. After 24 h incubation with drugs, luminescence values were measured using a Cytation5 plate reader (BioTek, Winooski, VT, USA). Standard curve and sample values were analyzed after subtraction of values from blank sample, and all statistical analyses were done by using Graphpad© Prism8 software (RRID:SCR_002798, GraphPad Software Inc., La Jolla, CA, www.graphpad.com). All experiment conditions had six technical repeats and experiments were repeated in six technical repeats at least for two times.

### 4.14. Statistical Analyses

Data from all studies were analyzed using a one-way analysis of variance (ANOVA) model to compare different ligand effects, a two-way-ANOVA model to compare time-dependent changes. All data were tested for normal distribution. Normally distributed data was analyzed using pairwise *t* tests with a Bonferroni correction to identify treatments that were significantly different from each other (* *p* < 0.05, ** *p* < 0.01, *** *p* < 0.001, **** *p* < 0.0001). For every main effect that was statistically significant at α = 0.05, pairwise t-tests were conducted to determine which ligand treatment levels were significantly different from each other. For these t-tests, the Bonferroni correction was employed to control experiment wise type I error rate at α = 0.05 followed by Bonferroni post hoc test. Data that were not normally distributed were analyzed using Mann–Whitney test for nonparametric data (* *p* < 0.05, ** *p* < 0.01, *** *p* < 0.0001). Statistical significance was calculated using GraphPad Prism for Windows.

### 4.15. Availability of Data and Materials

Gene expression data is submitted to GEO database under the accession number GSE112883 and will be available from the day of the acceptance of the manuscript.

## 5. Conclusions

Through our study, we evaluated combining SEL with current therapies to overcome therapy resistance in ER^+^ metastatic tumors. Using our molecular characterization methods in clinically relevant cell lines we analyzed how combined XPO1 targeting affects PGC1α level, therapy responsiveness, ERα signaling and tumor outcome, thereby evaluating the potential of its inhibition as a means to prevent metastatic tumor adaptation and therapy resistance. Overall, the results of our studies validated XPO1 as a target whose co-inhibition should enhance the effectiveness of therapies in metastatic ER^+^ breast tumors. ER-XPO1 and regulation of PGC1α have not been implicated in adaptation to metastatic environments and therapy resistance of ER^+^ metastatic tumors before. Given the need for better strategies for improving therapy response of relapsed ER^+^ tumors and avoid increasingly toxic therapy applied in this setting, our findings have great potential for uncovering the role of this protein and its targeting in reducing therapy resistance in recurrent ER^+^ tumors. In summary, nuclear export pathways have not previously been implicated or examined in metastatic tissue adaptation and therapy resistance, and given the need for better strategies for improving or maintaining therapy response of metastatic ER^+^ tumors, our studies carry high potential for uncovering the role of these pathways and their use in reducing deaths due to metastatic BCas.

## Supplementary Materials

The following are available online at https://www.mdpi.com/2072-6694/12/9/2397/s1, Figure S1: Isobologram analysis of 4-OHT, Fulv or Palb and SEL combinations in various cell lines; Figure S2: Dose-response of 4-OHT, Fulv or Palb with or without SEL; Figure S3: Colony formation assay for T47D-*ESR1*^Y537S^, T47D-*ESR1*^D538G^, HCC1500, MCF7 or T47D cells; Figure S4: Validation of glutamine dependence and related phenotypes in other cell lines; Figure S5: Validation of PGC1α-related biology.
