# Supplementary material for: Combined Targeting of Estrogen Receptor Alpha and Exportin 1 in Metastatic Breast Cancers
Source: Cancers (Basel). 2020 Aug 24;12(9):2397. doi: 10.3390/cancers12092397 (PMC7563274; doi:10.3390/cancers12092397)
Supplement: Supplementary file 1 [file cancers-12-02397-s001.pdf]

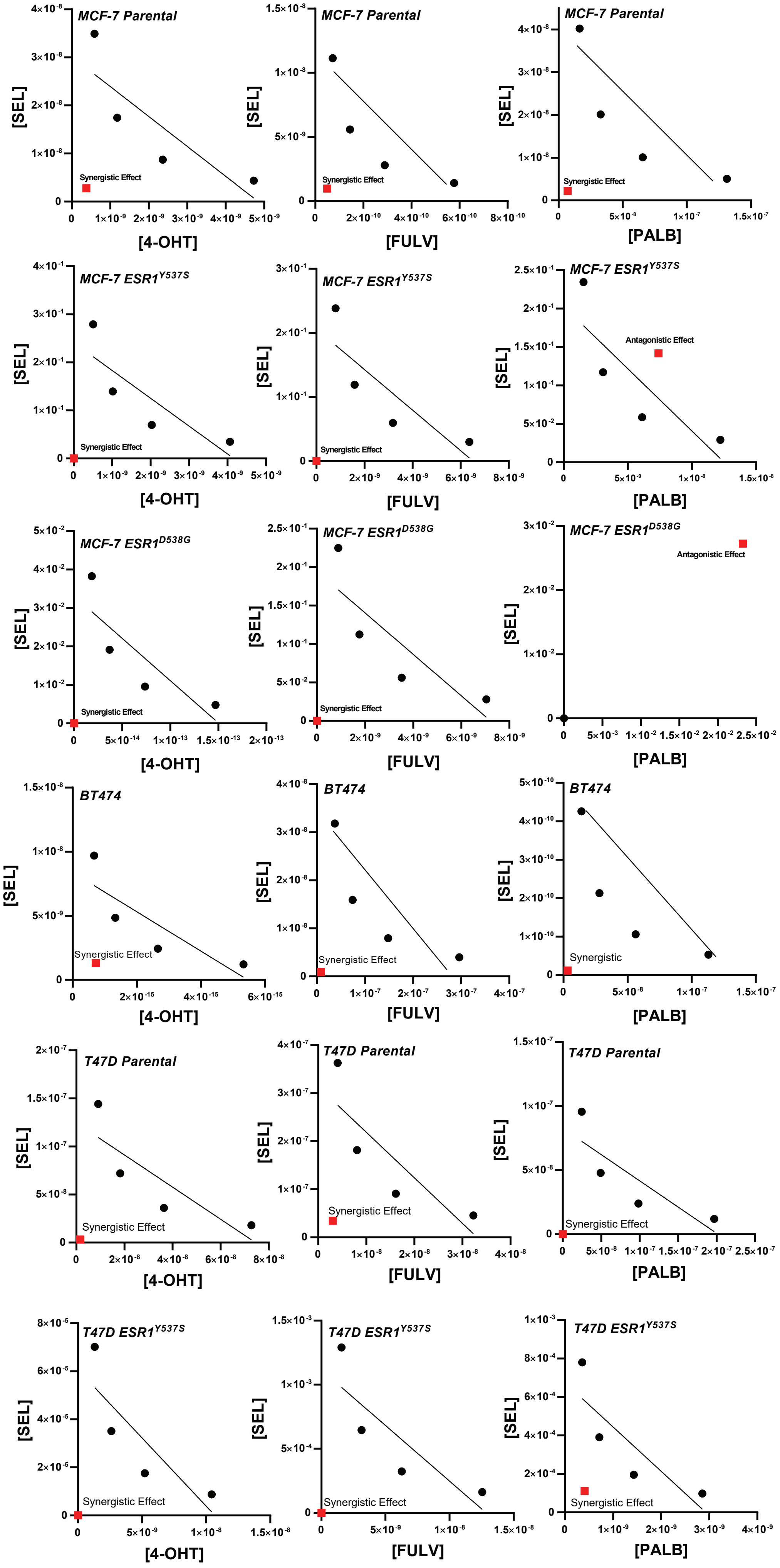

MCF-7 Parental
[SEL]
[4-OHT]
Synergistic Effect
MCF-7 Parental
[SEL]
[FULV]
Synergistic Effect
MCF-7 Parental
[SEL]
[PALB]
Synergistic Effect
MCF-7 $ESR1^{Y537S}$
[SEL]
[4-OHT]
Synergistic Effect
MCF-7 $ESR1^{Y537S}$
[SEL]
[FULV]
Synergistic Effect
MCF-7 $ESR1^{Y537S}$
[SEL]
[PALB]
Antagonistic Effect
MCF-7 $ESR1^{D538G}$
[SEL]
[4-OHT]
Synergistic Effect
MCF-7 $ESR1^{D538G}$
[SEL]
[FULV]
Synergistic Effect
MCF-7 $ESR1^{D538G}$
[SEL]
[PALB]
Antagonistic Effect
BT474
[SEL]
[4-OHT]
Synergistic Effect
BT474
[SEL]
[FULV]
Synergistic Effect
BT474
[SEL]
[PALB]
Synergistic
T47D Parental
[SEL]
[4-OHT]
Synergistic Effect
T47D Parental
[SEL]
[FULV]
Synergistic Effect
T47D Parental
[SEL]
[PALB]
Synergistic Effect
T47D $ESR1^{Y537S}$
[SEL]
[4-OHT]
Synergistic Effect
T47D $ESR1^{Y537S}$
[SEL]
[FULV]
Synergistic Effect
T47D $ESR1^{Y537S}$
[SEL]
[PALB]
Synergistic Effect

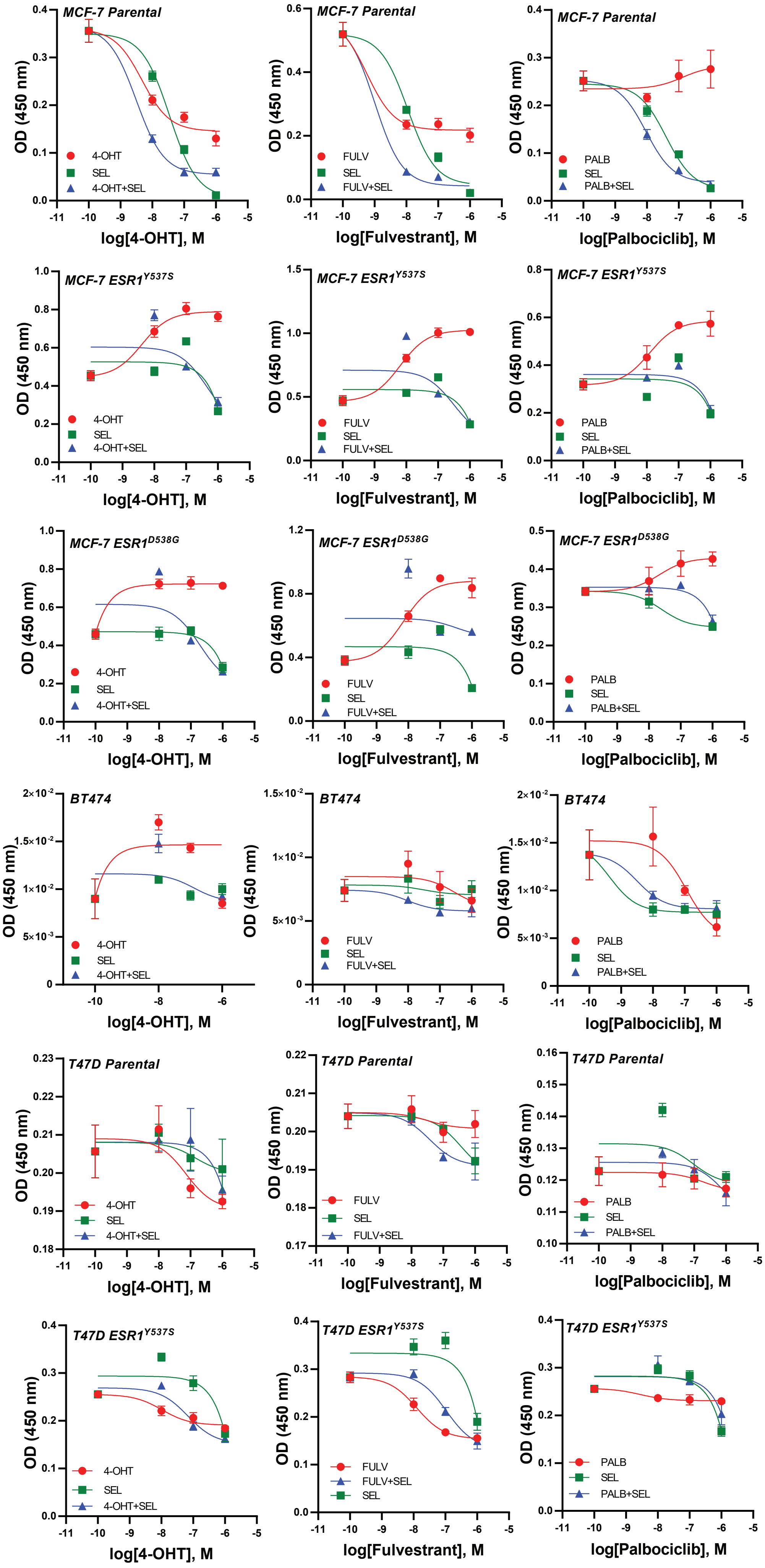

MCF-7 Parental
OD (450 nm)
4-OHT
SEL
4-OHT+SEL
log[4-OHT], M
MCF-7 Parental
OD (450 nm)
FULV
SEL
FULV+SEL
log[Fulvestrant], M
MCF-7 Parental
OD (450 nm)
PALB
SEL
PALB+SEL
log[Palbociclib], M
MCF-7 ESR1^{Y537S}
OD (450 nm)
4-OHT
SEL
4-OHT+SEL
log[4-OHT], M
MCF-7 ESR1^{Y537S}
OD (450 nm)
FULV
SEL
FULV+SEL
log[Fulvestrant], M
MCF-7 ESR1^{Y537S}
OD (450 nm)
PALB
SEL
PALB+SEL
log[Palbociclib], M
MCF-7 ESR1^{D538G}
OD (450 nm)
4-OHT
SEL
4-OHT+SEL
log[4-OHT], M
MCF-7 ESR1^{D538G}
OD (450 nm)
FULV
SEL
FULV+SEL
log[Fulvestrant], M
MCF-7 ESR1^{D538G}
OD (450 nm)
PALB
SEL
PALB+SEL
log[Palbociclib], M
BT474
OD (450 nm)
4-OHT
SEL
4-OHT+SEL
log[4-OHT], M
BT474
OD (450 nm)
FULV
SEL
FULV+SEL
log[Fulvestrant], M
BT474
OD (450 nm)
PALB
SEL
PALB+SEL
log[Palbociclib], M
T47D Parental
OD (450 nm)
4-OHT
SEL
4-OHT+SEL
log[4-OHT], M
T47D Parental
OD (450 nm)
FULV
SEL
FULV+SEL
log[Fulvestrant], M
T47D Parental
OD (450 nm)
PALB
SEL
PALB+SEL
log[Palbociclib], M
T47D ESR1^{Y537S}
OD (450 nm)
4-OHT
SEL
4-OHT+SEL
log[4-OHT], M
T47D ESR1^{Y537S}
OD (450 nm)
FULV
FULV+SEL
SEL
log[Fulvestrant], M
T47D ESR1^{Y537S}
OD (450 nm)
PALB
SEL
PALB+SEL
log[Palbociclib], M

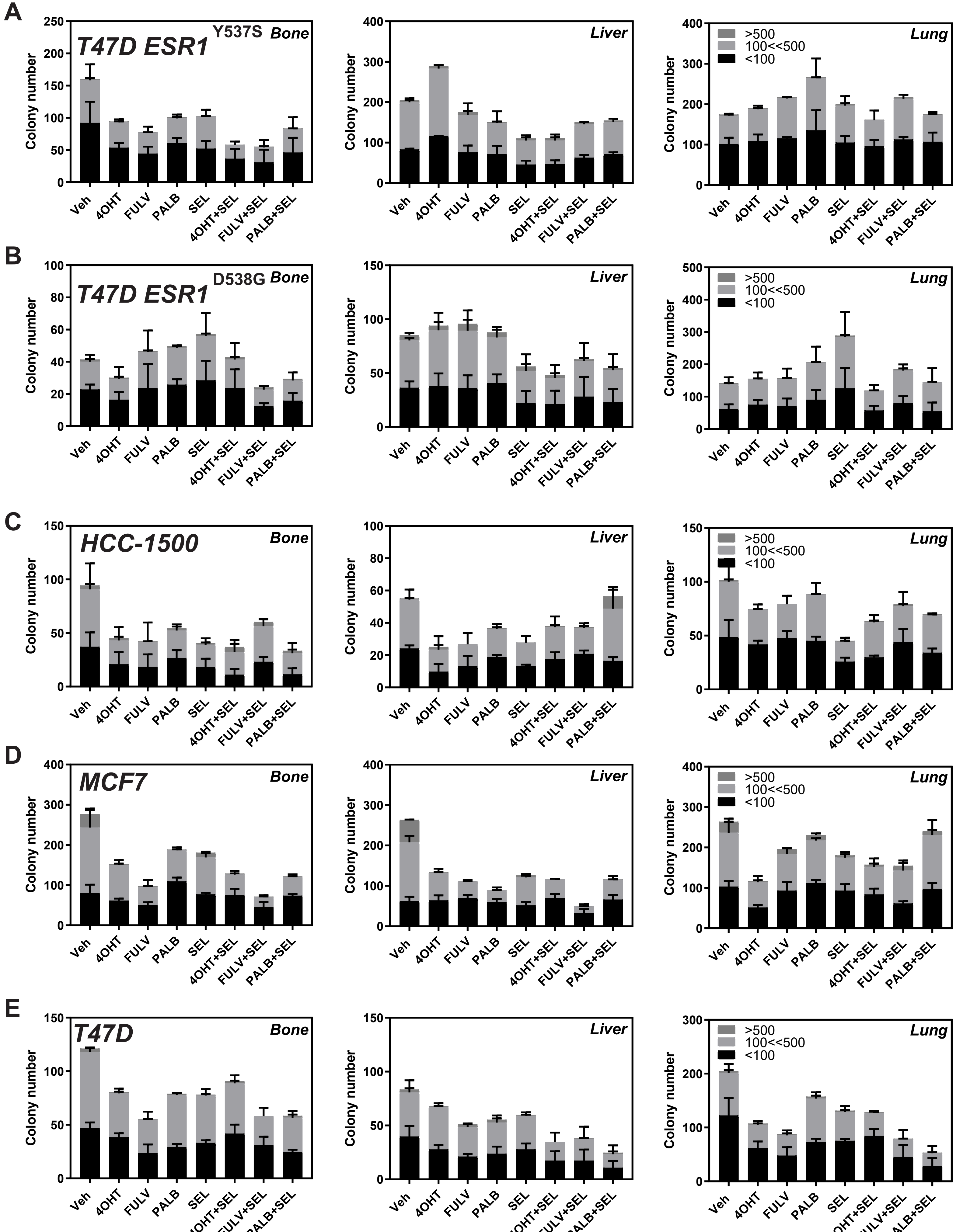

Supplementary Figure 3

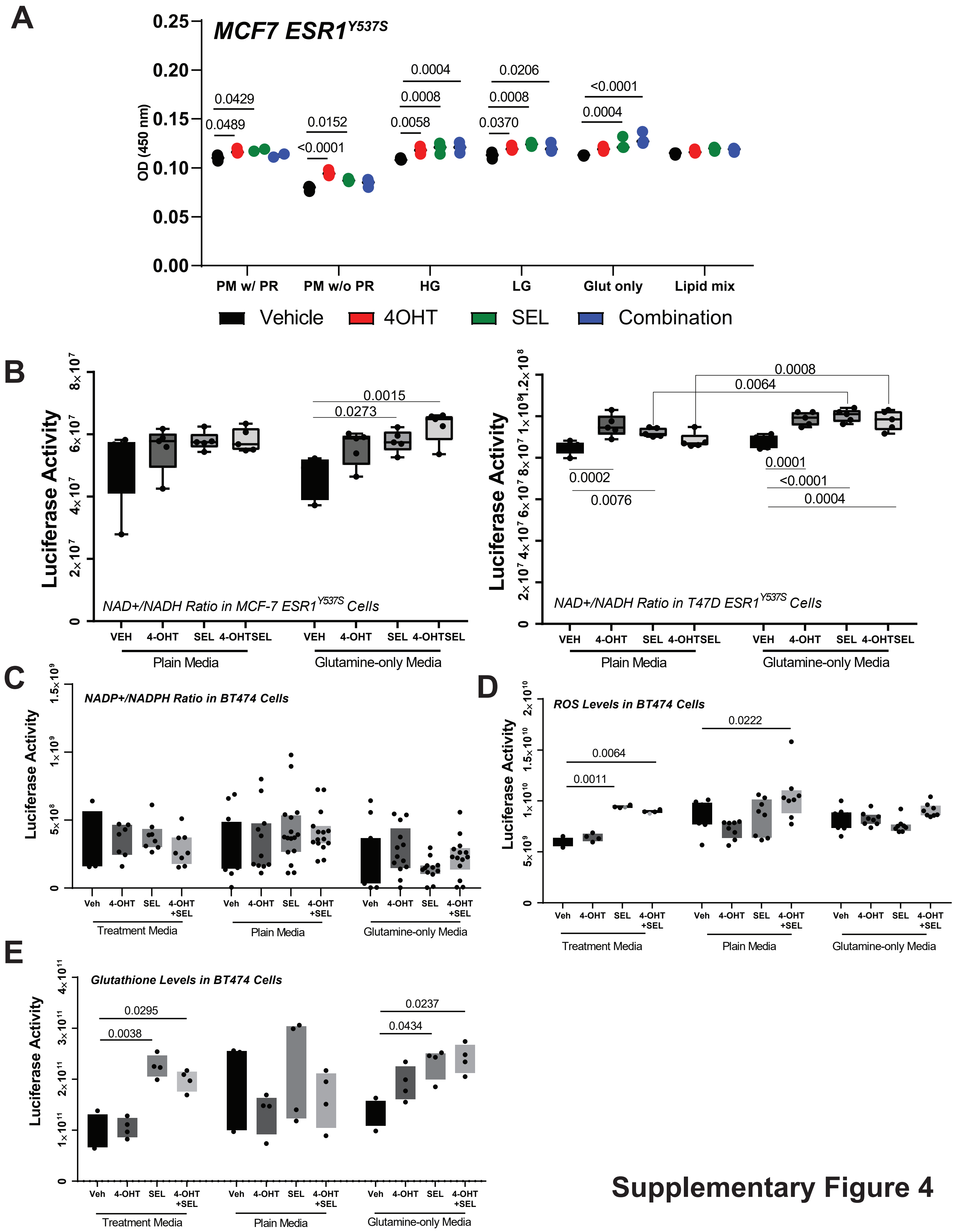

Supplementary Figure 4

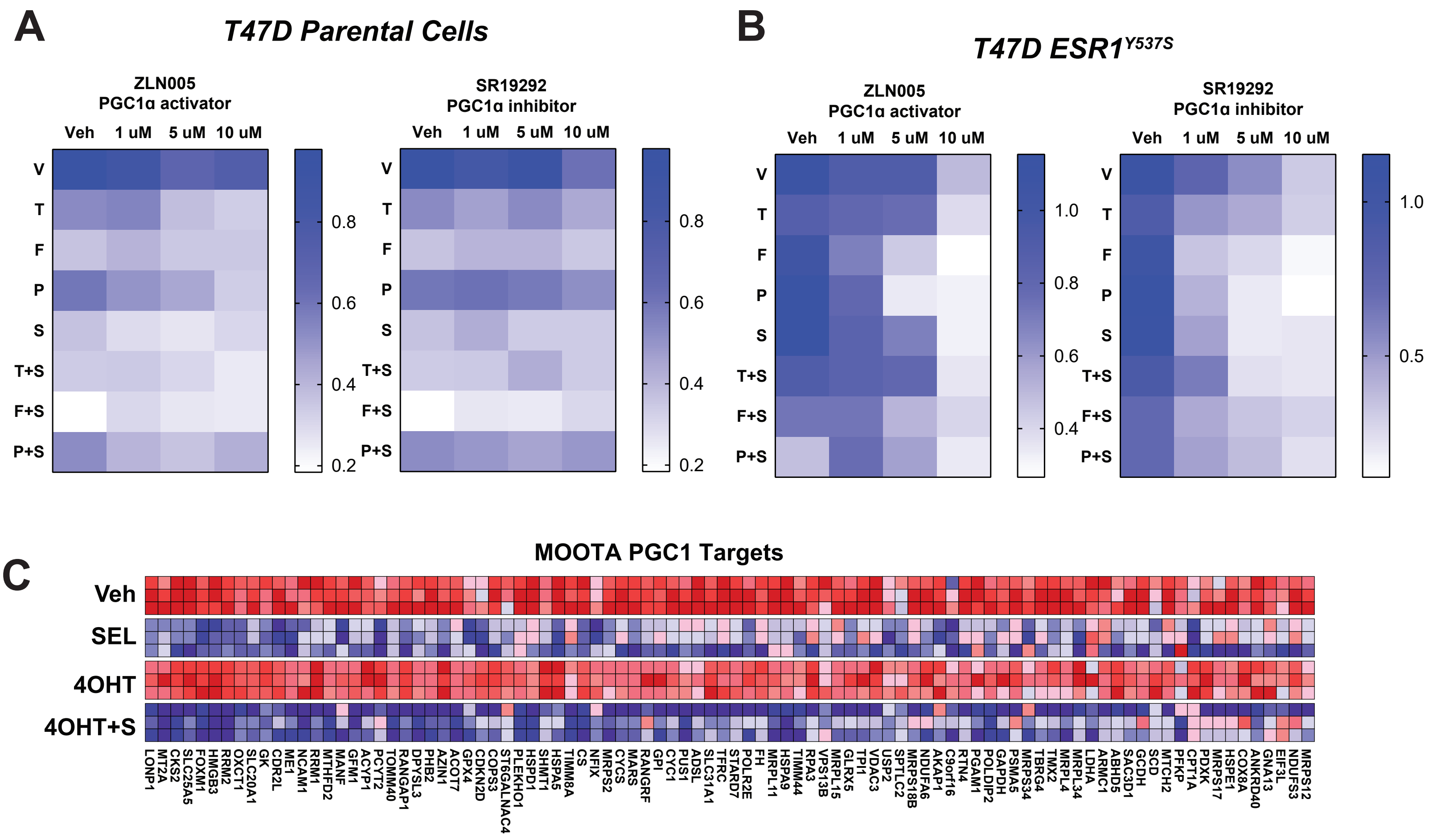

Supplementary Figure 5

Supplementary Figure 1. Isobologram analysis of 4-OHT, Fulv or Palb and SEL combinations in various cell lines

Supplementary Figure 2. Dose-response of 4-OHT, Fulv or Palb with or without SEL

Supplementary Figure 3. Colony formation assay for T47D-*ESR1*^{Y537S}, T47D-*ESR1D*^{538G}, HCC1500, MCF7 or T47D cells.

Supplementary Figure 4. A. Cell viability assay showing the effect of glutamine addition to the minimal media. MCF7-*ESR1*^{Y537S} cells were cultured at a density of $2x10^3$ cells/well in a 96-well plate and treated with 1 μM 4-OHT alone and in combination with 100 nM SEL in different minimal media conditions. **B.** NAD+/NADH assay in MCF7-*ESR1*^{Y537S}, MCF7-*ESR1D*^{538G}. $NADP^+$/NADPH ratios **C.**, reactive oxygen species (ROS) **D.** and Glutathione levels **E.** were quantified by using different luminescence-base assays. For each test kit, BT474 cells were seeded at a density of $2x10^3$ in 96-well plates and treated with 1 μM 4-OHT in combination with 100 nM SEL. Experimental statistics were analyzed by using GraphPad© Prism8 software. A two-way analysis of variance (ANOVA) model was used for statistical significance of treatment and values were presented as mean ± SEM from three independent experimental repeats. Significances were compared according to first measurement values for each treatment condition.

Supplementary Figure 5. Cell viability assays for T47D (**A.**) and T47D-*ESR1*^{Y537S} cells **B.** that were treated with 4-OHT or SEL combinations in the presence of ZLN005 or SR19292. **C.** MOOTHA PGC1 targets gene expression data from RNA-Seq experiment.
